# Electrocardiographic Alterations Combined with Hematological, Biochemical, and Metabolic Profiles Predict Prognosis in Kawasaki Disease

**DOI:** 10.3390/jcdd13060228

**Published:** 2026-05-27

**Authors:** Qirun Wang, Wenjuan Li, Jiaojiao Wan, Li Wei, Yuting Xia, Yimin Hua, Kaiyu Zhou, Di Qie, Weikai Li, Yifei Li

**Affiliations:** 1 Department of Pediatrics, Ministry of Education Key Laboratory of Women and Children’s Diseases and Birth Defects, West China Second University Hospital, Sichuan University, Chengdu 610041, China; qrwang1024@foxmail.com (Q.W.); baobao_8237@163.com (W.L.); vangelgel@163.com (J.W.); weiliisme@126.com (L.W.); summerrain68@126.com (Y.X.); nathan_hua@163.com (Y.H.); kaiyuzhou313@163.com (K.Z.); chachajuk@yeah.net (D.Q.); 2 School of Mathematics and Statistics, Chongqing Jiaotong University, Chongqing 400074, China

**Keywords:** ECG, Kawasaki disease, prognosis, machine learning, random forest algorithm

## Abstract

Objective: Kawasaki disease (KD) is characterized as an acute systemic vasculitis predominantly affecting young children, with coronary artery lesions (CALs) representing the most serious complication. Therapeutic resistance to intravenous immunoglobulin (IVIG) remains a significant clinical challenge. Consequently, numerous investigations have sought to identify predictive risk factors for IVIG resistance (IVIGR) and CAL development. Limited research has systematically evaluated the prognostic utility of electrocardiographic (ECG) parameters in KD outcome prediction. This study was therefore undertaken to assess the contributory value of ECG analysis in determining KD prognosis and therapeutic responses. Methods: This prospective cohort study enrolled 255 hospitalized children diagnosed with KD at West China Second University Hospital between July 2022 and December 2024. Initially, univariate analysis was performed to identify risk factors differentiating IVIGR from non-IVIGR patients and CAL from non-CAL patients. Statistically significant parameters were subsequently incorporated into machine learning analyses. Random forest algorithms were employed to construct predictive models based on the following: (1) complete blood count parameters, (2) biochemical and metabolic profiles, (3) electrocardiographic features, and (4) a comprehensive multimodal model integrating all parameters. These models generated feature importance scores, providing hierarchical rankings that quantified the relative contribution of each predictor to outcome prediction. Results: Univariate analysis demonstrated that alterations in hematological parameters, biochemical and metabolic profiles, and electrocardiographic features were significantly associated with therapeutic responses to IVIG and CAL development. Machine learning analysis revealed that ECG parameters individually contributed modest predictive weight for KD prognosis. However, the integration of ECG features into the comprehensive model substantially enhanced the discriminatory capacity, elevating the area under the curve (AUC) to 0.92 for CAL prediction. For IVIGR prediction, ECG-exclusive models demonstrated suboptimal performance in early disease management. Nevertheless, the multimodal integration of ECG with inflammatory and metabolic biomarkers achieved a comparable AUC of 0.92 for IVIGR prediction. Conclusions: This study establishes that ECG parameter alterations are significantly associated with CAL development and IVIGR in KD patients. Although ECG features demonstrate a limited independent predictive capacity compared to inflammatory and metabolic biomarkers, their integration into comprehensive predictive models substantially enhances discriminatory performance. These findings underscore the complementary value of electrocardiographic assessment in multimodal risk stratification strategies for KD management, supporting the clinical utility of ECG analysis as an adjunctive prognostic tool when combined with conventional laboratory parameters.

## 1. Introduction

Kawasaki disease (KD), also known as mucocutaneous lymph node syndrome, is an acute, self-limiting systemic vasculitis that predominantly affects small and medium arteries. KD can induce coronary artery damage, leading to vascular dilation and, in severe cases, aneurysm formation, myocardial infarction, heart failure, myocarditis, pericarditis, valvular inflammation accompanied by pericardial effusion, and even coronary artery rupture resulting in hemopericardium and sudden death [1]. Essentially, all KD-related mortality and morbidity stems from cardiac complications. Consequently, the prognosis of KD is critically dependent on the severity of coronary artery lesions (CALs) [2]. Accumulating evidence suggests that KD has supplanted rheumatic heart disease as the leading cause of acquired heart disease in children [3,4,5]. The precise etiology and pathogenesis of KD remain incompletely understood, although the disease typically manifests in children aged 6 months to 5 years [3]. Epidemiological observations suggest that KD may have an infectious etiology, as evidenced by distinct seasonal patterns and sex-specific differences in certain geographic regions, and especially given that the prevalence of KD reduced during the COVID-19 pandemic after facial mask administration [6,7]. Regarding its pathogenesis, marked systemic immune activation occurs during the acute phase of KD, characterized by an elevated number of inflammatory mediators; this sustained inflammatory response is postulated to be a critical mechanism underlying coronary artery injury. It is well established that the combination of oral aspirin (ASA) and high-dose intravenous immunoglobulin (IVIG) (2 g/kg) achieves clinical remission in the majority of KD patients [1,8]. However, approximately 10–20% of patients exhibit persistent fever following initial IVIG treatment, a condition termed IVIG resistance (IVIGR) [9,10]. This phenomenon likely reflects ongoing inflammatory activity and potential coronary artery damage, which adversely affect therapeutic efficacy and clinical outcomes.

Consequently, the development of prediction systems and prognostic models for KD represents a critical frontier in current KD research. Extensive research exists on risk factors and predictive models for both IVIGR and CALs in KD. Multiple studies have identified potential predictive biomarkers, including age, total bilirubin (TB), erythrocyte sedimentation rate (ESR), aspartate aminotransferase (AST), C-reactive protein (CRP) [9], neutrophil percentage (Neu%), N-terminal pro-B-type natriuretic peptide (NT-proBNP) [11,12], serum ferritin (sF), serum acid sphingomyelinase (ASM), calcineurin (CaN), serum amyloid A (SAA), serum interleukin-17A (IL-17A), interferon-gamma (IFN-γ), interleukin-6 (IL-6), pentraxin-3 (PTX3) [13,14], BECN1, and LC3II m-RNA [15], all of which have demonstrated associations with IVIGR and CALs [16,17,18]. However, the predictive performance of these biomarkers exhibits substantial heterogeneity across different countries, geographic regions, ethnic populations, and treatment protocols. For instance, Norazah Zahari et al. identified leukocytosis and fingertip changes as independent risk factors for IVIGR [19], whereas studies conducted in Chinese populations found these indicators to lack predictive value [18]. Most existing studies on IVIGR in KD have primarily relied on pretreatment clinical and laboratory data. In this context, the work of Kobayashi et al. is particularly informative: they developed a seven-variable logistic regression model incorporating hematologic and inflammatory parameters measured both before IVIG administration and 24 h after the initial infusion in a cohort of 546 KD patients [11]. In addition, potential protective factors against IVIGR have been reported. For example, Wang et al. found that serum albumin (ALB) may be protective, with hypoalbuminemia (≤2.9 g/dL) associated with an increased risk of IVIG treatment failure [20]. Notably, most of these investigations have focused on blood-based biomarkers. Although individual parameters often show limited discriminatory ability for predicting IVIGR, composite indices derived from routine laboratory variables may improve clinical applicability, including the systemic immune-inflammation index (SII) [21], neutrophil–lymphocyte ratio (NLR), platelet–lymphocyte ratio (PLR) [22,23], and prognostic nutritional index (PNI) [24]. Nevertheless, findings for the same indicators remain inconsistent across studies and have not yet achieved broad consensus. Beyond the prediction of IVIGR, the formula to assess the risk of CALs has also been extended. And several parameters, including the PNI, the ratio of CRP to albumin, and the NLR, have been calculated to establish the association between potential inflammation parameters and the incidence of CALs [23,25]. Moreover, the genetic susceptibility to CALs of all KD patients has been considered a major contributor [7,26]. Current studies largely focus on developing complex prediction systems to identify severe inflammatory activity associated with IVIGR or CALs. However, early markers reflecting cardiac alterations or myocardial injury have received comparatively limited attention and have not been systematically evaluated.

Accordingly, several biomarkers of myocardial injury have been examined in prior studies. Nevertheless, the predictive value of electrocardiography (ECG) for IVIGR and CALs in KD, particularly ECG vector signals, remains insufficiently characterized. Therefore, the present study further investigated risk factors by integrating laboratory biomarkers, relevant clinical variables, and ECG features. We first applied conventional statistical methods to identify potential high-risk predictors. We then used a random forest machine learning approach to construct prediction models for IVIGR and CALs based on four feature domains: complete blood count, biochemical parameters, ECG features, and the combination of laboratory and ECG data [27]. The random forest algorithm enables the derivation of feature importance scores for each variable. The development of these models, particularly the quantification of predictive importance for individual variables, may facilitate more precise identification of clinically significant predictors.

## 2. Methods

### 2.1. Patient Population

This study was approved by the Ethics Committee of West China Second University Hospital of Sichuan University in accordance with the Declaration of Helsinki (NO. 2021-069). Written informed consent was obtained from all subjects. The clinical data were obtained from patients diagnosed with KD at West China Second Hospital of Sichuan University from July 2022 to December 2024. Overall, 255 children (152 males and 103 females) with an average age of 3.18 years (0.18 to 11.60 years) suffering from KD were included, out of whom 68 cases underwent a treatment procedure with IVIGR (26.7% of total), while 187 cases were considered sensitive to IVIG administration. While CALs appeared in 51 cases (20% of total) during the acute or subacute phase of KD, 204 cases were normal. This was a single-center prospective cohort study conducted at the hospital; all the included research cases met the inclusion and exclusion criteria.

### 2.2. Inclusion and Exclusion Criteria

We recruited candidates for further analysis using the following inclusion criteria: (1) All patients should meet the diagnostic criteria for a complete or incomplete diagnosis of KD as recommended by the AHA (2017) [9], and the diagnosis should be confirmed by two physicians, including complete or incomplete KD diagnosis, treatment, and long-term management. (2) All patients should have received IVIG and aspirin treatment within 10 days of onset, and a complete routine blood test and serum biochemical analysis should have been completed within 2 days prior to treatment with IVIG. (3) All the patients should be younger than 14. (4) Echocardiographic screening should be performed for coronary artery injuries or aneurysms in the acute or subacute phase. (5) ECG should be recorded before IVIG administration with vector calculations. (6) There should be a complete collection of basic information, clinical presentation, laboratory test results, treatment process, and short-term follow-up (6 months) results. Exclusion criteria included the following: (1) Children who received non-standard IVIG treatment or used corticosteroids or any other immunosuppressive drugs before or at the same time as the initial IVIG treatment; (2) Patients with recurrent Kawasaki disease; (3) Patients with congenital heart disease; (4) Patients who received antiplatelet or anticoagulant drugs before KD onset; (5) Patients with inherited cardiomyopathy or arrhythmias; (6) Patients with suspected myocarditis; and (7) Patients with other vasculitis or autoimmune diseases.

In addition, we identified the manifestation of IVIGR and CALs according to the AHA (2017) guidelines [9]. IVIGR has been identified as the condition where after standard full-dose IVIG treatment within 10 days of fever onset, neither the patient’s fever subsides within 36 h (temperature is still over 38 °C) nor their temperature returns to normal; instead, their fever returns within 2–7 days or 2 weeks, accompanied by at least one KD clinical feature [5,15]. CALs were recognized on the basis of coronary artery internal lumen diameters ≥ 3 mm in children <5 years of age or ≥4 mm in children ≥5 years of age; a diameter exceeding 1.5 times the diameter of the adjacent segment; or the Z-score of the coronary artery lumen exceeding 2 [9,18].

### 2.3. Therapeutic and Follow-Up Procedures

All patients with Kawasaki disease were treated with high-dose intravenous immunoglobulin (2 g/kg given as a single intravenous infusion) combined with 30–50 mg/kg/day high-dose aspirin. Those with recrudescent or persistent fever for ≥36 h after the first dose of intravenous immunoglobulin were treated with a second dose of 2 g/kg intravenous immunoglobulin. Methylprednisolone (30 mg/kg/day for 3 consecutive days) followed by oral prednisone tapered over 7 days was considered after the second intravenous immunoglobulin administration. The 1st day of fever was defined as the 1st day of illness onset. Intravenous immunoglobulin resistance was defined as persistent or recurrent fever (oral temperature of ≥38.0 °C) or other clinical signs of Kawasaki disease for at least 36 h but not >7 days after the initial intravenous immunoglobulin infusion. The patients were discharged from the hospital when their temperature had remained normal for >48 h and hematological examination showed normal values. Follow-up was started from hospital discharge, and all the involved patients were required to revisit the hospital for echocardiographic evaluations at 2 weeks, 1 month, 2 months, 3 months, 6 months, and 12 months, which was documented from the end of the subacute phase. The coronary artery aneurysms, which regressed within one month, were considered early regression, while the lesions that returned after one month were considered delayed regression.

### 2.4. Data Collection

Collected medical information included demographic data and laboratory results. The demographic data included hospitalization date, hospitalization number, sex, age, and body mass index (BMI). Laboratory test results included complete blood count, biochemical indicators (liver function, serum lipids, renal function, cardiac enzymes, and electrolytes, etc.), and electrocardiogram (including vector cardiogram data) at the acute stage of KD (usually no more than 10 days). The collection of all clinical data and follow-up was performed by experienced physicians. All clinical data had supporting electronic medical records, follow-up databases, and original registration documents. ECG was carried out using a MECG-1000 (Medex tech., Beijing, China) machine with a standard 12-lead, 500 Hz sampling frequency and was recorded after the graph had been stable for more than 10 s. The ECG reading values and calculated values, including vector assessments, peak voltages, duration, intervals, and QT interval correction were calculated instantly using the machine’s software, documented, and included for analysis. The data used in analysis and prediction were recorded once per patient at the time of initial evaluation, before treatment initiation.

### 2.5. Vectorcardiogram (VCG) Analysis

**Figure 1:**
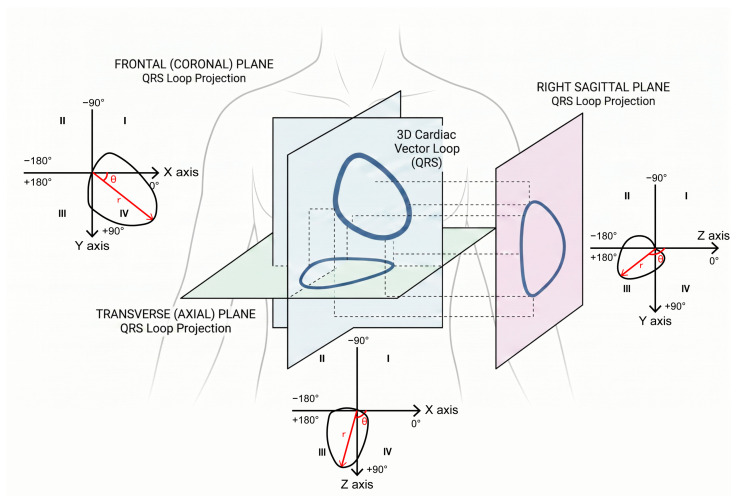
Schematic illustration of the vectorcardiogram and its planar projections. The cardiac vector loop is projected onto the frontal (coronal), transverse (axial), and right sagittal planes (blue circles). Dashed lines connect each of its two-dimensional planar projections, illustrating the spatial relationship of orthogonal planar representations. Frontal plane is defined by the horizontal axis (positive leftward) and the vertical axis (positive downward). Transverse plane is defined by the horizontal axis (positive leftward) and the vertical axis (positive posterior). Right sagittal plane is defined by the horizontal axis (positive posterior) and the vertical axis (positive downward). Quadrant I, II, III, and IV’s boundaries are defined at 0°, 90°, 180°, and −90° (−180°) relative to the positive horizontal axis, respectively. Parts of Figure 1 have been created using BioRender’s AI generate function (app.biorender.com) and artificially revised; the shape of cardiac vector loops and dashed lines of loops in three planes are only for demonstration and may differ from reality.

The vectorcardiogram was reconstructed from the standard 12-lead electrocardiogram (ECG), and was recorded using conventional limb and precordial electrode placements. Orthogonal X, Y, and Z leads were mathematically derived from the 12-lead signals by build using the transformation algorithm of the machine’s software. The frontal plane was constructed by plotting X (horizontal axis, positive leftward) against Y (vertical axis, positive downward); the transverse plane by plotting X (horizontal) against Z (vertical, positive posterior); and the right sagittal plane by plotting Z (horizontal) against Y (vertical). Schematic illustration of the VCG and its planar projections are illustrated in Figure 1.

The P wave, QRS wave, and T wave loop were projected into each plane. To quantify the spatial distribution of heart depolarization, the area enclosed by these loops within each quadrant of the frontal, transverse, and sagittal planes was computed. The loop was expressed in polar coordinates as the instantaneous magnitude *r* and angle *θ*, and the area in a given quadrant was obtained via numerical integration $S=\frac{1}{2}\int r^{2}d\theta$ over the angular interval defining that quadrant, using the trapezoidal rule. Areas were expressed in mV^2^, and the areas of the I, II, III, and IV quadrants in the frontal plane, transverse plane, and sagittal plane in VCG were calculated (abbreviated to frontal I area, etc.).

The R-T angle was defined as the angle between the maximal QRS vector and the maximal T vector, in the same projected plane. The maximal QRS vector was identified as the vector from the origin to the farthest point on the QRS loop, and its angle α_QRS_ was measured from the positive horizontal axis. The maximal T vector and its angle α_T_ were determined analogously. The R-T angle was calculated as the absolute difference ∣α_QRS_ − α_T_∣, which was then reduced to the acute/obtuse interval 0–180° (if the difference exceeded 180°, it was subtracted from 360°). This calculation was performed separately for the frontal, transverse, and right sagittal planes (abbreviated to frontal R-T angle, etc.).

### 2.6. Statistical Analysis

Patients were stratified into statistical groups based on the occurrence of IVIGR or CALs, where the IVIG-resistant and IVIG-sensitive groups, as well as the CAL and normal coronary artery groups, were treated as independent cohorts for comparative analysis. The minimum sample size required to achieve adequate statistical power was calculated using Ssize software (version 1.86.0, repository from Bioconductor Release 3.23), with the parameters set according to anticipated effect sizes derived from the previously published literature, ensuring that the study was sufficiently powered to detect clinically meaningful differences while minimizing the risk of Type I and Type II errors. All statistical analyses were performed using SPSS version 22.0 software (SPSS Inc., Chicago, IL, USA). The normality of continuous variables was assessed using the Shapiro–Wilk test prior to analysis. Continuous variables conforming to a normal distribution are expressed as mean ± SD and were compared between groups using independent-sample *t*-tests. Categorical variables are presented as frequencies and percentages, and group differences were evaluated using the Chi-square test or Fisher’s exact test where applicable. All reported *p*-values are two-tailed, and a *p*-value of less than 0.05 was considered statistically significant.

### 2.7. Machine Learning Analysis

This study employed the random forest [27] classification algorithm for prediction. Separate models were constructed based on four distinct feature sets: complete blood count, blood biochemistry, ECG, and a comprehensive set integrating all indicators. Instead of relying on the direct classification output from each individual model, a weighted voting fusion strategy was adopted. Specifically, the vote counts from each base random forest model were used as their outputs. An optimal combination of weights for these outputs was identified through a grid search, with the average accuracy from five-fold cross-validation serving as the evaluation metric. Once the optimal weights were determined, all data were fed into random forest’s sub-models, and their outputs were aggregated via weighted fusion to produce the final prediction. This ensemble design leverages the strengths of the different data types, thereby enhancing the overall predictive performance. A comparative analysis of the five individual models was ultimately conducted to elucidate the significance of ECG features in predicting outcomes for children with KD. To address the issue of class imbalance, data splitting into sub-models was strictly maintained in the original class distribution, ensuring that the training and test sets were representative of the overall dataset. For model interpretation, the features were ranked by their importance scores [28]. A key advantage of the random forest model is its inherent ability to rank predictive features by importance, which provides valuable insights for clinical decisions. Each model computed importance scores for all features that were ranked in descending order. Typically, the mean decrease in accuracy [29] (MDA) method is used to measure feature importance. The main steps are presented in Table 1.

The underlying assumption of this “mean decrease in accuracy” method is that if feature d has a significant impact on the prediction result, the number of correctly classified samples will significantly decrease after introducing substantial noise. Typically, the importance score is a positive value. However, as shown in the calculation formula in Step 7, the importance score for a specific feature $d^{\prime }$ can be close to zero or even negative. An importance score close to zero indicates that adding perturbation to feature $d^{\prime }$ has no effect on the prediction outcome. This suggests that the feature is irrelevant to the target variable. In the model we constructed, each feature was scored, and a higher score indicated a more significant impact on the prognosis, which can provide guidance for clinical decisions.

### 2.8. LASSO Logistic Regression and Cross-Validation

Because our dataset comprised over 80 predictor variables with inherent collinearity (e.g., among blood cell indices, liver/renal function tests, and ECG parameters) and a limited number of outcome events, conventional multivariable logistic regression was vulnerable to overfitting, variance inflation, and complete or quasi-complete separation. To address these issues, we applied the least absolute shrinkage and selection operator (LASSO) logistic regression [30]. LASSO introduces an L1-penalty term into the log-likelihood function, which shrinks regression coefficients toward zero and forces some coefficients to become exactly zero, thereby performing automatic variable selection. The penalty is governed by a tuning parameter λ: larger values of λ yield sparser models with fewer retained predictors, while λ = 0 recovers the standard logistic regression. The optimal λ was chosen to balance model fit and complexity.

To obtain unbiased estimates of model performance and to avoid over-optimism, we used 10-fold cross-validation. Specifically, the dataset was randomly partitioned into 10 equally sized subsets. In each iteration, one subset served as the validation set while the remaining nine were used for model training. The LASSO model was fitted on the training data across a range of λ values, and the predicted probabilities for the validation samples were recorded. This procedure was repeated until every subset had been used once for validation. The cross-validated predictions were then pooled to compute the area under the receiver operating characteristic curve (AUC), net reclassification improvement (NRI), and integrated discrimination improvement (IDI). The λ that minimized the cross-validated deviance λ_min_ was selected for generating prediction scores, while the more conservative λ that was one standard error larger λ_1se_ was used to identify the most parsimonious set of independent predictors. All analyses were performed using the glmnet package in R (version 5.0) [31].

## 3. Results

### 3.1. Identification of Potential Risk Factors in Determining CALs

This study enrolled a total of 255 pediatric patients diagnosed with KD, comprising 204 cases without CALs and 51 cases with CALs. The non-CAL cohort consisted of 114 males and 90 females, with a mean age of 3.25 years (range: 2 months to 11 years). The CAL group included 38 males and 13 females, with a mean age of 2.9 years (range: 3 months to 11 years and 7 months). Initially, we sought to identify potential risk factors associated with CAL development, with statistical analyses presented in Table 2. Comparative analysis revealed that patients with CALs exhibited a significantly elevated body mass index (BMI) compared to the non-CAL group (16.51 ± 1.81 vs. 15.72 ± 1.94, *p* = 0.009), suggesting that BMI constitutes a risk factor for coronary artery involvement. Complete blood count analysis demonstrated statistically significant differences in hemoglobin (HGB), neutrophil percentage (Neu%), and the red cell distribution width (RDWC). Specifically, the CAL group exhibited lower hemoglobin levels (106.07 vs. 111.85 g/L, *p* = 0.005) and a reduced neutrophil percentage (64.00 ± 15.59% vs. 68.70 ± 15.07%, *p* = 0.048), while the RDWC significantly increased (13.40 ± 1.27% vs. 13.00 ± 0.93%, *p* = 0.040). These findings may be attributed to the erythrocyte destruction that is secondary to severe systemic inflammatory responses. Biochemical and lipid profile analyses identified prealbumin, C-reactive protein (CRP), alanine aminotransferase (ALT), serum potassium, and serum magnesium as statistically significant risk factors for CALs. Notably, CRP (89.48 ± 37.56 vs. 69.63 ± 44.92 mg/L, *p* = 0.004) and ALT (73.98 ± 69.35 vs. 44.44 ± 44.26 U/L, *p* = 0.006) levels were markedly elevated in the CAL group, whereas prealbumin levels were significantly reduced (47.49 ± 15.85 vs. 59.34 ± 33.85 mg/L, *p* = 0.001). Electrocardiographic analysis revealed that the S wave amplitude in lead V5 (SV5) significantly increased in the CAL group compared to the non-CAL group (0.70 ± 0.50 vs. 0.47 ± 0.30 mV, *p* = 0.003), demonstrating statistical significance.

### 3.2. Machine Learning Revealed Different Contributions in Determining CALs

**Figure 2:**
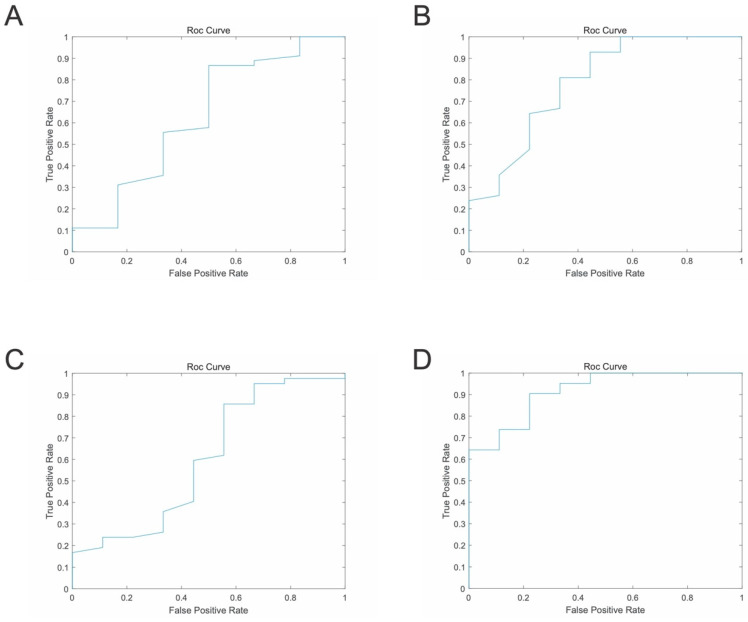
Receiver operating characteristic (ROC) curves with corresponding area under ROC (AUC) of four models in CAL, derived from five-fold cross-validation. False Positive Rate, 1–specificity; True Positive Rate, sensitivity. (**A**) For blood tests model, AUC = 0.63; (**B**) for blood biochemical and lipids analysis model, AUC = 0.78; (**C**) for ECG evaluation model, AUC = 0.60; and (**D**) for comprehensive model, AUC = 0.92.

We constructed random forest models utilizing complete blood count (CBC) parameters, biochemical markers, individual ECG data, as well as a comprehensive model integrating all available data. Model performance was evaluated using the mean accuracy and area under the receiver operating characteristic curve (AUC) derived from five-fold cross-validation. The AUC values for the four predictive models are illustrated in Figure 2. Comparative analysis revealed that models employing a single data domain as a predictive feature yielded AUC values of 0.63 (CBC), 0.78 (biochemical), and 0.60 (ECG). While these models demonstrated a modest predictive capability, their discriminatory performance remained suboptimal. In contrast, the comprehensive model incorporating all indicators achieved an AUC of 0.92, demonstrating a substantially superior discriminatory capacity.

**Figure 3:**
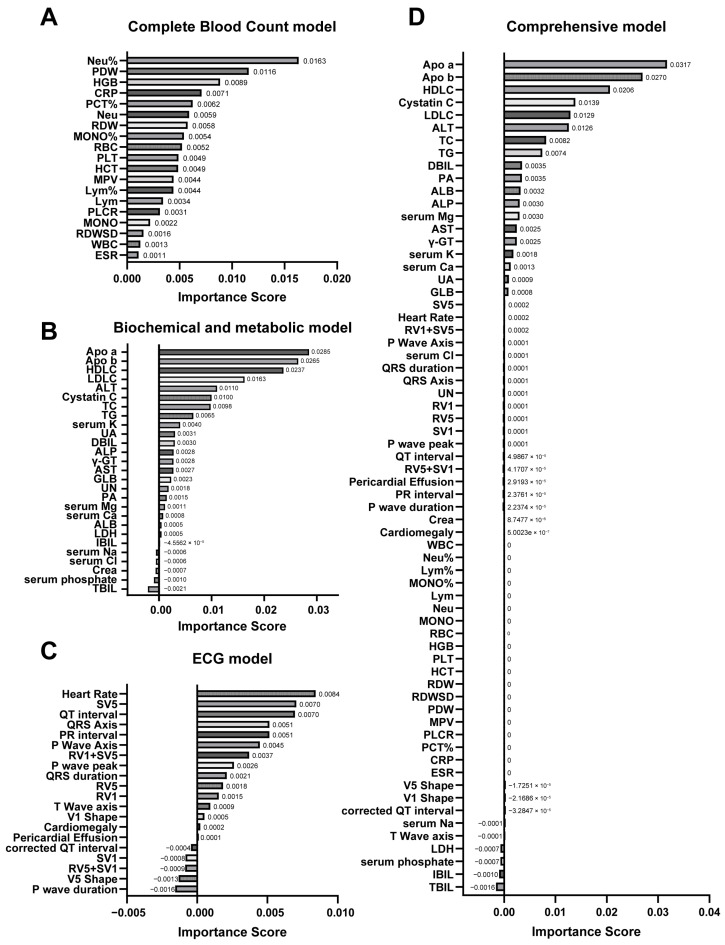
Importance scores derived from machine learning models for predicting coronary artery lesions (CALs). Variables are ranked in descending order of importance, with higher scores indicating greater influence on model prediction. All variable importance scores are normalized to sum to 1 within each model. (**A**) Hematological model (complete blood count parameters). (**B**) Biochemical and metabolic model (serum lipids and biochemistry markers). (**C**) Electrocardiographic (ECG) model. (**D**) Comprehensive model combining all laboratory and ECG variables. Abbreviations for variables: WBC, white blood cell; Neu%, neutrophil percentage; Lym%, lymphocyte percentage; Mono%, monocyte percentage; Lym, absolute lymphocyte count; Neu, absolute neutrophil count; Mono, absolute monocyte count; RBC, red blood cell; HGB, hemoglobin; PLT, platelet; HCT, hematocrit; RDWC, red cell distribution width; RDWSD, red cell distribution width standard deviation; PDW, platelet distribution width; MPV, mean platelet volume; PLCR, platelet–large contrast ratio; PCT%, procalcitonin percentage; CRP, C-reactive protein; ESR, erythrocyte sedimentation rate; ALT, alanine aminotransferase; AST, aspartate aminotransferase; TB, total bilirubin; DBIL, direct bilirubin; IDIL, indirect bilirubin; ALB, albumin; GLB, globulin; γ-GT, glutamyl transpeptidase; LDH, lactate dehydrogenase; PA, prealbumin; ALP, alkaline phosphatase; UN, urea nitrogen; Crea, creatinine; UA, uric acid; serum K, serum potassium; serum Na, serum sodium; serum Cl, serum chloride; serum Ca, serum calcium; serum Mg, serum magnesium; TC, total cholesterol; TG, triacylglycerol; HDLC, high-density lipoprotein cholesterol; LDLC, low-density lipoprotein cholesterol; Apoa, apolipoprotein A; Apob, apolipoprotein B; P wave peak, maximum voltage of P wave among the leads; QRS peak, maximum voltage of P wave among the leads; RV1, R-wave amplitude in V1; RV5, R-wave amplitude in V5; SV1, S-wave amplitude in V1; and SV5, S-wave amplitude in V5.

Given the limited sample size of this study, we implemented five-fold cross-validation to maximize the utilization of available cases and rigorously assess both the accuracy and robustness of the predictive models. The mean five-fold cross-validation accuracy rates for the CBC model, biochemical model, ECG model, and comprehensive model were 0.82, 0.87, 0.80, and 0.87, respectively. A key advantage of the random forest algorithm is its capacity to quantify the relative importance of individual predictors, enabling feature ranking that provides valuable insights for clinical decision-making. Figure 3 depicts the feature importance distributions across the four predictive models.

### 3.3. Identification of Potential Risk Factors in Determining IVIGR

Among the 255 KD patients enrolled in this study, 68 cases (26.7%) demonstrated IVIGR, comprising 37 males and 31 females with a mean age of 3.43 years (range: 3 months to 11 years and 7 months). The IVIGS group consisted of 187 cases, including 115 males and 72 females, with a mean age of 3.09 years (range: 2 months to 11 years). Therefore, we conducted statistical analyses to identify potential risk factors associated with IVIGR, with results presented in Table 3.

Complete blood count analysis revealed that the neutrophil percentage, lymphocyte percentage, absolute neutrophil count (ANC), and absolute lymphocyte count (ALC) constituted significant risk factors for IVIGR. The IVIGR group exhibited a significantly elevated ANC (10.72 ± 4.56 vs. 8.80 ± 3.84 × 10^9^/L, *p* = 0.001) and neutrophil percentage (73.21 ± 16.71% vs. 66.09 ± 14.91%, *p* = 0.001), while demonstrating a reduced ALC (2.27 ± 1.39 vs. 3.25 ± 2.76 × 10^9^/L, *p* = 0.006) and lymphocyte percentage (17.06 ± 11.24% vs. 24.48 ± 12.28%, *p* = 0.001). Although IVIG therapy typically attenuates inflammatory responses in small- and medium-sized arteries in KD patients, IVIGR cases appear to exhibit more severe or persistent inflammation, manifesting as pronounced neutrophilia with compensatory lymphopenia—findings consistent with previous reports [11,32].

Biochemical analysis identified multiple statistically significant markers: elevated markers in IVIGR included total bilirubin (TB), direct bilirubin (DBIL), indirect bilirubin (IBIL), blood urea nitrogen (UN), uric acid (UA), and triglycerides (TG), while decreased markers in IVIGR were albumin (ALB), high-density lipoprotein cholesterol (HDL-C), and apolipoprotein A (ApoA). In IVIGR patients, elevated TB and DBIL levels coupled with decreased ALB concentrations suggest hepatic involvement, mirroring patterns observed in CAL patients. The elevated UN and UA levels in the IVIGR cohort indicate compromised renal function. Furthermore, alterations in lipid profiles—including decreased HDL-C and Apo A—suggest dysregulated lipid metabolism, potentially associated with hepatic dysfunction and a systemic inflammatory status. Serum magnesium demonstrated a modest but significant reduction (0.85 ± 0.08 vs. 0.82 ± 0.06 mmol/L, *p* = 0.035).

ECG parameters revealed significant differences in the QRS duration (77.09 ± 7.83 vs. 74.22 ± 9.70 ms, *p* = 0.018), QRS axis (81.79 ± 29.55° vs. 71.89 ± 29.27°, *p* = 0.018), and SV5 amplitude (0.62 ± 0.47 vs. 0.46 ± 0.32 mV, *p* = 0.012) among IVIGR patients. These alterations likely reflect coronary artery involvement, ongoing inflammatory processes, and vascular endothelial injury characteristic of KD’s pathophysiology.

### 3.4. Machine Learning Revealed Different Contributions in Determining IVIGR

**Figure 4:**
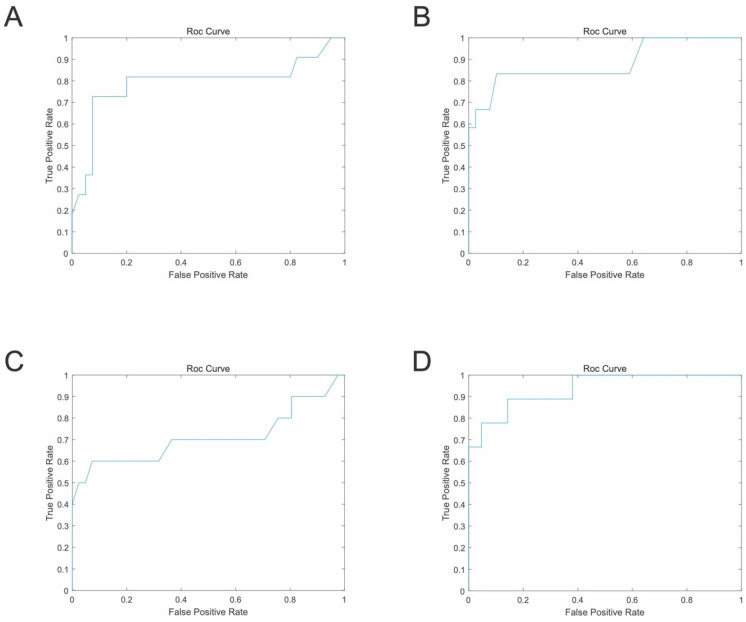
Receiver operating characteristic (ROC) curves with corresponding area under ROC (AUC) of four models in IVIGR, derived from five-fold cross-validation. False Positive Rate, 1–specificity; True Positive Rate, sensitivity. (**A**) for blood tests model, AUC = 0.79; (**B**) for blood biochemical and lipids analysis model, AUC = 0.88; (**C**) for ECG evaluation model, AUC = 0.71; and (**D**) for comprehensive model, AUC = 0.94.

**Figure 5:**
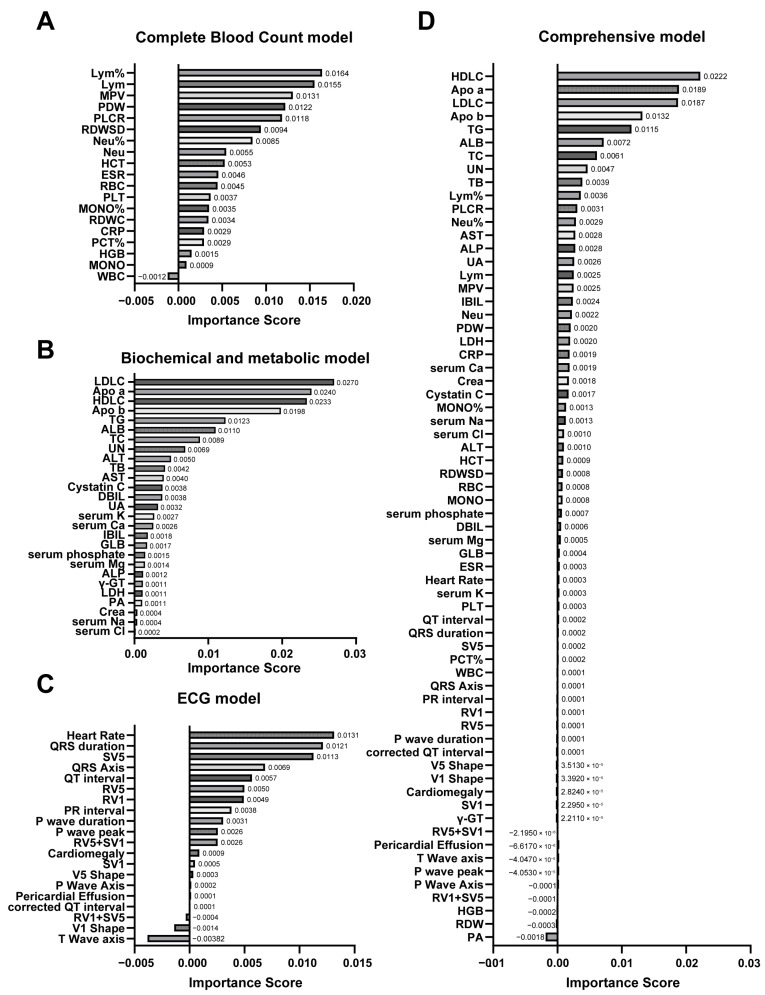
Importance scores derived from machine learning models for predicting intravenous immunoglobulin resistance (IVIGR). Variables are ranked in descending order of importance, with higher scores indicating greater influence on model prediction. All variable importance scores are normalized to sum to 1 within each model. (**A**) Hematological model (complete blood count parameters). (**B**) Biochemical and metabolic model (serum lipids and biochemistry markers). (**C**) Electrocardiographic (ECG) model. (**D**) Comprehensive model combining all laboratory and ECG variables. Abbreviations for variables: WBC, white blood cell; Neu%, neutrophil percentage; Lym%, lymphocyte percentage; Mono%, monocyte percentage; Lym, absolute lymphocyte count; Neu, absolute neutrophil count; Mono, absolute monocyte count; RBC, red blood cell; HGB, hemoglobin; PLT, platelet; HCT, hematocrit; RDWC, red cell distribution width; RDWSD, red cell distribution width standard deviation; PDW, platelet distribution width; MPV, mean platelet volume; PLCR, platelet–large contrast ratio; PCT%, procalcitonin percentage; CRP, C-reactive protein; ESR, erythrocyte sedimentation rate; ALT, alanine aminotransferase; AST, aspartate aminotransferase; TB, total bilirubin; DBIL, direct bilirubin; IDIL, indirect bilirubin; ALB, albumin; GLB, globulin; γ-GT, glutamyl transpeptidase; LDH, lactate dehydrogenase; PA, prealbumin; ALP, alkaline phosphatase; UN, urea nitrogen; Crea, creatinine; UA, uric acid; serum K, serum potassium; serum Na, serum sodium; serum Cl, serum chloride; serum Ca, serum calcium; serum Mg, serum magnesium; TC, total cholesterol; TG, triacylglycerol; HDLC, high-density lipoprotein cholesterol; LDLC, low-density lipoprotein cholesterol; Apoa, apolipoprotein A; Apob, apolipoprotein B; P wave peak, maximum voltage of P wave among the leads; QRS peak, maximum voltage of P wave among the leads; RV1, R-wave amplitude in V1; RV5, R-wave amplitude in V5; SV1, S-wave amplitude in V1; and SV5, S-wave amplitude in V5.

Previous investigations have established a strong association between IVIGR and increased susceptibility to CAL development. Consequently, we constructed random forest predictive models for IVIGR using the aforementioned five clinical data domains (Figure 4). The complete blood count model, biochemical model, ECG model, and comprehensive model yielded AUC values of 0.79, 0.88, 0.71, and 0.94, respectively, with corresponding five-fold cross-validation accuracy rates of 0.80, 0.83, 0.76, and 0.85. These metrics demonstrate that the comprehensive model exhibited optimal predictive performance, followed by the biochemical model, though the performance differential between these two models was relatively modest. Models constructed exclusively from ECG parameters demonstrated a moderate discriminatory capacity but exhibited comparatively limited predictive efficacy. Figure 5 depicts the feature importance distributions across the four predictive models for IVIGR.

### 3.5. Multivariable LASSO Logistic Regression with Cross-Validation

To evaluate whether ECG parameters provide incremental predictive value beyond laboratory markers, we adopted a logistic regression analysis. As the dataset contained over 80 predictor variables with a substantial number of inter-correlations, but a moderate number (*n* = 255) of outcome events, conventional multivariable logistic regression was precluded by overfitting and complete separation. We therefore employed LASSO-penalized logistic regression with 10-fold cross-validation to evaluate the independent and incremental predictive value of ECG parameters beyond laboratory markers for both CALs and IVIGR. Missing data were imputed using random forest imputation, and all performance estimates were derived from cross-validated predictions to avoid optimism.

**Figure 6:**
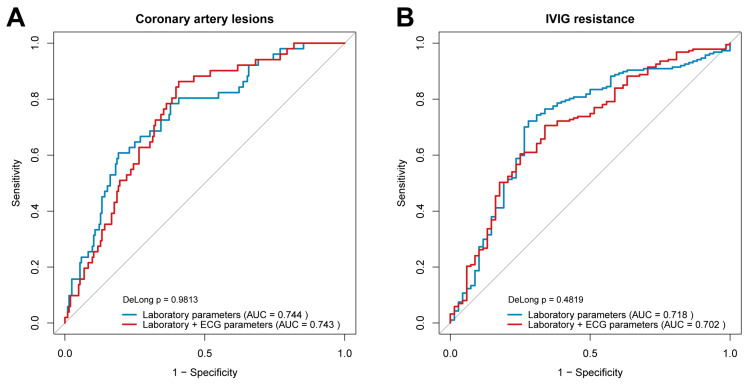
Cross-validated receiver operating characteristic (ROC) curves evaluating the incremental predictive value of electrocardiographic parameters. (**A**) Coronary artery lesions (CALs). (**B**) Intravenous immunoglobulin resistance (IVIGR). In each panel, the blue curve represents the laboratory-only model, and the red curve represents the laboratory + ECG model. The diagonal line indicates the reference line for random classification. *p*-values were calculated using the DeLong test.

For CALs, the laboratory-only model yielded a cross-validated AUC of 0.744, which increased marginally to 0.747 after adding ECG parameters (Figure 6A, ΔAUC = +0.004, DeLong *p* = 0.897). The continuous net reclassification improvement (NRI) and the integrated discrimination improvement (IDI) are shown in Table 4. Notably, the NRI for non-events was 0.196 (*p* = 0.004), indicating that ECG parameters significantly improved the correct downward reclassification of patients without CALs. Two ECG variables, the heart rate (coefficient = 0.0004) and SV5 voltage (coefficient = 0.5632), were retained in the LASSO model, confirming their independent association after full adjustment for laboratory covariates.

For IVIGR, the laboratory-only model achieved an AUC of 0.718, whereas the addition of ECG variables resulted in an AUC of 0.702 (Figure 6B, ΔAUC = −0.016, DeLong *p* = 0.482). In contrast to CALs, the overall NRI and IDI reached statistical significance, driven mainly by an improved specificity component (decrease in predicted probability for non-events). Only the SV5 voltage was retained in the IVIGR model, with a negative coefficient (coefficient = −0.049), suggesting an inverse association with the risk of immunoglobulin resistance.

Collectively, these results indicate that in a traditional linear multivariable framework, ECG parameters provide limited incremental discriminatory power (negligible ΔAUC for CALs, slightly negative for IVIGR). By contrast, the comprehensive machine learning model that included ECG parameters will benefit significantly from more variables.

## 4. Discussion

With the advent of machine learning algorithms, the early prediction of CALs and IVIGR has become increasingly feasible and clinically relevant. Despite the modest standalone predictive performance of ECG parameters, electrocardiography retains substantial clinical value in KD management. As a non-invasive, readily accessible, and cost-effective diagnostic modality, ECG facilitates real-time cardiovascular monitoring without radiation exposure or sedation requirements—particularly advantageous in pediatric populations.

Comparative analysis of feature importance scores in the CAL prediction model revealed hierarchical differences between single-domain and comprehensive models. In the ECG-exclusive model, the top three predictors were heart rate, SV5 amplitude, and QT interval. Conversely, in the comprehensive model, the leading ECG parameters—SV5, heart rate, and RV1 + SV5—ranked substantially lower than biochemical markers. This redistribution likely reflects inter-feature interactions and correlations; however, the precise mechanistic relationships remain obscured by the inherent “black-box” nature of machine learning algorithms. Both the biochemical and comprehensive models achieved identical five-fold cross-validation accuracy rates of 0.87 for CAL prediction. Notably, the predominant predictive features in the comprehensive model were derived from lipid metabolism and hepatic function parameters, corroborating findings from univariate CAL analysis. However, while the biochemical model yielded an AUC of 0.78, the comprehensive model achieved an AUC of 0.92. This substantial improvement suggests that although ECG parameters individually contribute modest predictive weight, their integration enhances the model’s discriminatory capacity and accentuates the predictive significance of hepatic biomarkers through synergistic interactions.

Univariate analysis of IVIGR revealed significantly elevated TB and DBIL levels, accompanied by decreased ALB concentrations in the IVIGR cohort. As previously discussed regarding hepatic involvement in CAL patients, three principal mechanisms may account for hepatic dysfunction in KD. According to the existing literature, reduced ALB levels may result from impaired hepatic synthesis secondary to liver injury [20], or alternatively, from increased vascular permeability induced by systemic vasculitis, facilitating ALB extravasation into the extravascular compartment. The concurrent elevation of DBIL suggests more severe hepatocellular damage or pronounced ALB leakage in IVIGR patients. Elevated blood UN and UA levels in the IVIGR group indicate compromised renal function. Although renal impairment is relatively uncommon in KD, its presence typically signifies severe disease and substantially increases IVIGR risk. Clinicians should maintain heightened vigilance for Kawasaki disease shock syndrome (KDSS) in such patients.

Alterations in IBIL, TG, HDL-C, and ApoA reflect dyslipidemia associated with hepatic dysfunction and a systemic inflammatory status. Severe inflammatory conditions frequently precipitate lipid metabolic derangements, and our findings regarding HDL-C and ApoA align with numerous previous KD investigations. The underlying mechanism by which KD disrupts lipid homeostasis likely involves endothelial dysfunction and diminished lipoprotein lipase activity, resulting in attenuated HDL-C synthesis and accelerated Apo A catabolism [33,34]. These metabolic perturbations may serve as biomarkers of disease severity and therapeutic resistance.

Analysis of feature importance in the IVIGR prediction model revealed that biochemical parameters, particularly lipid metabolism markers, constituted the predominant predictors in the comprehensive model, including HDL-C, apolipoprotein A, LDL-C, apolipoprotein B, total cholesterol, and total bilirubin. These were followed by additional biochemical indicators encompassing hepatic enzymes, uric acid, blood urea nitrogen, AST, and ALP, as well as hematological parameters including lymphocyte and neutrophil percentages. Notably, complete blood count parameters demonstrated greater predictive utility for IVIGR compared to for CALs, where their contribution approached negligible values. This differential performance aligns with the superior AUC achieved for IVIGR prediction (0.79 versus 0.63 for CALs), potentially reflecting the more pronounced relationship between IVIGR—a manifestation of severe systemic inflammation—and immune cell dynamics. The existing literature presents inconsistent findings regarding these biomarkers, with identical parameters yielding divergent levels of statistical significance across studies. Currently, no universally validated risk factors for CALs or IVIGR have been established, limiting their direct clinical applicability. Machine learning-based predictive models address this limitation by providing hierarchical rankings of feature importance, revealing that biochemical markers possess a superior predictive capacity for both CALs and IVIGR. This phenomenon likely stems from hepatic dysfunction and lipid metabolic derangements induced by the severe inflammatory cascade and endothelial injury characteristic of KD pathogenesis.

KD is an acute, self-limiting systemic vasculitis predominantly affecting the coronary arteries. The extent of coronary artery involvement critically influences long-term patient prognosis. ECG abnormalities, including alterations in QRS duration, QRS axis deviation, and S wave amplitude variations (such as SV5), may reflect subclinical myocardial involvement, conduction disturbances, or ventricular strain secondary to coronary inflammation. Furthermore, serial ECG monitoring allows for the dynamic assessment of disease progression and therapeutic responses, complementing echocardiographic evaluation [35]. The enhanced predictive accuracy observed when ECG data were incorporated into comprehensive models underscores the importance of multiparametric risk stratification strategies. ECG parameters may capture distinct pathophysiological dimensions—such as electrical remodeling and myocardial stress—that are not fully represented by biochemical or hematological markers alone [36,37]. This synergistic effect suggests that integrated diagnostic approaches, combining ECG with laboratory biomarkers and clinical features, optimize risk prediction and may facilitate personalized therapeutic decision-making. Therefore, this study aimed to evaluate the predictive utility of electrocardiographic parameters in identifying coronary artery involvement in KD patients. However, our findings diverged from initial expectations: ECG parameters did not emerge as primary predictors of either CALs or IVIGR. Instead, lipid profiles and hepatic function markers demonstrated a superior predictive capacity. Notably, while ECG data alone exhibited limited discriminatory power, their integration into comprehensive multimodal models significantly enhanced the overall predictive accuracy [38,39].

Previous investigations have demonstrated a positive correlation between elevated BMI and cardiovascular disease risk. In KD, higher BMI has been associated with increased CAL susceptibility at equivalent disease stages and a prolonged recovery duration [40,41,42]. Regarding the relationship between peripheral blood cell parameters and CALs, the existing literature reports associations with elevated WBC and PLT counts, though consensus remains elusive. Our study revealed significantly reduced HGB levels in the CAL cohort, potentially attributable to erythrocyte destruction secondary to severe systemic inflammation. RDW, a marker of erythrocyte size heterogeneity traditionally employed in anemia evaluation, has demonstrated statistical associations with cardiovascular morbidity and mortality [43]. The elevated RDW observed in our CAL group corroborates erythrocyte disruption resulting from inflammatory processes or cardiovascular injury. Markedly elevated CRP levels reflect substantial inflammatory mediator release during the acute disease phase. Increased ALT and decreased PA in the CAL group indicate hepatic dysfunction—an increasingly prevalent KD complication. Current evidence attributes KD-associated hepatic injury to three principal mechanisms: (1) the pharmacological effects of pre-diagnostic medications; (2) direct pathogen-mediated hepatotoxicity in infection-triggered cases; and (3) hepatic vasculitis and oxidative stress induced by severe inflammatory responses. Notably, the Egami scoring system for KD severity assessment incorporates ALT as a key predictive parameter [1,44,45]. Serum potassium and magnesium concentrations were elevated in the CAL group while remaining within their physiological ranges, likely reflecting internal milieu perturbations. Despite their modest magnitude, these alterations achieved statistical significance, suggesting potential clinical relevance in risk stratification.

Numerous publications have identified the spatial QRS-T angle as a prognostic electrocardiographic marker; however, its clinical utility remains constrained by age-, sex-, and population-specific variations in reference ranges [46]. Nevertheless, these electrocardiographic alterations demonstrate that coronary artery damage in KD results in detectable changes in cardiac electrical activity. The underlying pathophysiological mechanism likely involves coronary artery dilatation and aneurysm formation—hallmark features of KD vasculopathy. These structural abnormalities promote hemodynamic disturbances, including reduced flow velocity and turbulence, which predispose patients to thrombosis. Additionally, the intense inflammatory milieu characteristic of KD induces endothelial dysfunction and hypercoagulability, further facilitating thrombus formation or precipitating coronary vasospasm, ultimately resulting in myocardial ischemia. Long-term follow-up studies indicate that approximately 23% of KD patients with giant coronary artery aneurysms develop acute myocardial infarction, with 6% succumbing to thrombotic coronary occlusion [47]. Radionuclide myocardial perfusion imaging has corroborated the presence of myocardial ischemia in these patients [48,49]. These findings substantiate that coronary pathology in KD significantly influences cardiac electrophysiology, rendering ECG-based assessment of coronary involvement clinically feasible. Given the documented myocardial ischemia and hypoxia in KD, electrocardiographic evaluation represents a viable approach for assessing myocardial injury severity.

Our analysis identified several ECG parameters demonstrating statistical associations with IVIGR and CALs, indicating detectable cardiac electrical abnormalities during acute KD. Electrocardiography offers distinct advantages of accessibility, rapidity, and non-invasiveness; thus, its utility in predicting adverse KD outcomes would confer substantial clinical value [50]. However, machine learning analysis revealed that these parameters possessed lower predictive weight than initially anticipated. Importantly, ECG integration enhanced the overall model accuracy, with multimodal models incorporating complete blood count, biochemical profiles, and electrocardiographic data, achieving optimal performance in both accuracy and discriminatory capacity.

## 5. Conclusions

The early identification and prognostic prediction of IVIGR and CAL-complicated KD patients represent critical clinical imperatives. This study systematically identified risk factors for CALs and IVIGR, subsequently developing prediction models using machine learning algorithms based on multimodal clinical, laboratory, and ECG data. The results demonstrated that the alterations in ECG parameters were associated with CALs and IVIGR in KD. Unfortunately, the evaluations of ECG provided limited contributions in determining treatment outcomes and the prognosis of KD compared to inflammation and metabolic indicators. Nonetheless, the identification of ECG alterations definitely promoted the predictive value in the comprehensive model, demonstrating the effects of ECG analysis in KD management.

## Figures and Tables

**Table 1:** The dominant steps of random forest model analysis.

Step 1	A set of sample subsets, denoted as $\chi_{1},\cdots ,\chi_{M}$, was generated from the initial training dataset $\chi =\left(X,Y\right)$ through bootstrap sampling (bagging).
Step 2	A decision tree $T_{1}$ was trained using the sample subset $\chi_{1}$; the out-of-bag (OOB) samples for this decision tree were marked as $L_{1}^{oob}$.
Step 3	Decision tree $T_{1}$ was used to make predictions but without using the OOB samples $L_{1}^{oob}$. The number of correctly classified samples was recorded as $R_{1}^{obb}$.
Step 4	For each feature numbered d=1,2,⋯,D, the *d*-th feature in the OOB sample $L_{1}^{obb}$ was randomly permuted in sequence. This process generated new permuted OOB sample series, denoted as $L_{1,1}^{obb},\cdots ,L_{1,D}^{obb}$.
Step 5	The decision tree $T_{1}$ was used to make predictions on each of these new permuted OOB samples. The corresponding numbers of correctly classified samples were recorded as $R_{1,1}^{obb},\cdots ,R_{1,D}^{obb}$.
Step 6	Steps 1 through 5 were repeated for the remaining sample subsets $\chi_{2},\cdots ,\chi_{M}$. This yielded the counts of correctly classified samples before and after permutation for all trees and features: $\left(R_{2}^{obb},R_{2,1}^{obb},\cdots ,R_{2,D}^{obb}),\cdots ,(R_{M}^{obb},R_{M,1}^{obb},\cdots ,R_{M,D}^{obb}\right)$.
Step 7	The importance score for the *d*-th feature was calculated using the formula: $P_{d}=\frac{1}{M}\sum_{m=1}^{M}\left(R_{m}^{obb}-R_{m,d}^{obb}\right)$.
Step 8	All importance scores from feature 1 to feature D were collected.

**Table 2:** Univariate analysis of risk factors for CALs.

Factors	Non-CAL (*n* = 204)	CAL (*n* = 51)	*p*-Value
Age	3.25 ± 1.98	2.90 ± 2.40	0.281
Male	114	28	0.461
Female	90	23	
BMI (kg/m^2^)	15.72 ± 1.94	16.51 ± 1.81	0.009
***Blood tests***			
WBC (10^9^/L)	13.38 ± 4.23	13.66 ± 4.8	0.675
Neu (10^9^/L)	9.35 ± 4.05	9.1 ± 4.42	0.698
Neu%	68.7 ± 15.07	64 ± 15.59	0.048
Lym (10^9^/L)	2.93 ± 2.64	3.2 ± 1.87	0.499
Lym%	22.05 ± 11.94	24.38 ± 14.17	0.233
Mono (10^9^/L)	0.93 ± 0.98	0.97 ± 1.18	0.774
Mono%	6.61 ± 5.00	5.77 ± 2.35	0.246
HGB (g/L)	111.85 ± 10.26	106.07 ± 12.98	0.005
RBC (10^12^/L)	4.20 ± 0.42	4.26 ± 0.52	0.419
RDWC (%)	13.00 ± 0.93	13.40 ± 1.27	0.040
RDWSD (fl)	38.20 ± 2.43	38.00 ± 2.94	0.629
HCT (%)	33.65 ± 2.75	32.99 ± 3.54	0.152
PLT (10^9^/L)	342.09 ± 107.78	334.26 ± 123.32	0.654
PLCR (%)	22.61 ± 7.64	22.7 ± 7.9	0.945
PCT%	0.33 ± 0.09	0.34 ± 0.09	0.457
PDW (fL)	10.58 ± 2.02	10.77 ± 2.44	0.557
MPV (fL)	9.80 ± 0.95	9.76 ± 0.97	0.833
CRP (mg/L)	69.63 ± 44.92	89.48 ± 37.56	0.004
ESR (mm/h)	58.84 ± 26.73	61.67 ± 28.21	0.506
***Biochemistry and lipids***			
ALT (U/L)	44.44 ± 44.26	73.98 ± 69.35	0.006
AST (U/L)	59.45 ± 101.4	59.41 ± 54.78	0.998
GLB (g/L)	25.64 ± 15.94	24.38 ± 7.9	0.587
ALB (g/L)	39.49 ± 5.07	39.01 ± 4.22	0.539
TB (μmol/L)	11.41 ± 14.97	9.29 ± 9.29	0.337
DBIL (μmol/L)	5.98 ± 11.6	4.73 ± 7.56	0.469
IDIL (μmol/L)	5.02 ± 3.34	4.45 ± 2.29	0.252
ALP (U/L)	196.66 ± 73.9	195.64 ± 66.08	0.928
LDH (U/L)	301.04 ± 101.9	326.33 ± 159.76	0.290
Crea (μmol/L)	27.01 ± 6.77	26.15 ± 6.49	0.413
Cystatin C (mg/L)	1.40 ± 7.56	0.90 ± 0.16	0.639
γ-GT (U/L)	53.98 ± 69.9	59.3 ± 64.15	0.623
UN (mmol/L)	3.26 ± 1.20	3.32 ± 1.07	0.770
UA (μmol/L)	224.64 ± 71.35	223.81 ± 72.07	0.941
PA (μmol/L)	59.34 ± 33.85	47.49 ± 15.85	0.001
TC (mmol/L)	3.26 ± 0.54	3.16 ± 0.40	0.219
TG (mmol/L)	1.35 ± 0.42	1.29 ± 0.26	0.358
HDLC (mmol/L)	0.73 ± 0.25	0.71 ± 0.20	0.377
LDLC (mmol/L)	2.50 ± 1.43	2.35 ± 0.46	0.470
Apoa (g/L)	0.81 ± 0.21	0.76 ± 0.15	0.105
Apob (g/L)	0.75 ± 0.14	0.78 ± 0.11	0.165
Serum Na (mmol/L)	135.56 ± 3.00	135.71 ± 2.90	0.751
Serum Ca (mmol/L)	2.23 ± 0.16	2.26 ± 0.11	0.340
Serum Mg (mmol/L)	0.83 ± 0.08	0.87 ± 0.06	0.006
Serum K (mmol/L)	4.09 ± 0.50	4.29 ± 0.50	0.014
Serum Cl (mmol/L)	101.48 ± 7.68	102.25 ± 2.89	0.486
Serum phosphate (mmol/L)	1.29 ± 0.26	1.3 ± 0.24	0.907
***ECG analysis***			
PR interval (ms)	123.49 ± 16.53	124.78 ± 15.87	0.616
P wave duration (ms)	81.64 ± 11.53	81.08 ± 8.82	0.748
P wave peak (mV)	0.10 ± 0.07	0.10 ± 0.04	0.655
QT interval (ms)	287.66 ± 40.02	281.37 ± 40.72	0.320
Corrected QT interval (ms)	401.22 ± 27.03	399.84 ± 36.04	0.763
QRS duration (ms)	74.77 ± 9.35	75.86 ± 9.17	0.456
QRS peak (mV)	1.67 ± 2.77	1.34 ± 0.55	0.493
Transverse I area	39.19 ± 21.2	42.46 ± 24.37	0.437
Transverse II area	4.28 ± 5.93	1.87 ± 1.58	0.001
Transverse III area	9.70 ± 9.52	10.63 ± 9.46	0.611
Transverse IV area	47.34 ± 23	45.08 ± 27.3	0.622
Frontal I area	73.87 ± 18.47	73.31 ± 20.78	0.877
Frontal II area	11.58 ± 12.22	12.58 ± 14.55	0.683
Frontal III area	10.5 ± 12.26	8.32 ± 11.63	0.353
Frontal IV area	4.33 ± 7.68	5.71 ± 11.63	0.404
Frontal R-T angle	20.85 ± 31.53	36.03 ± 39.79	0.019
Transverse R-T angle	43.74 ± 42.12	47.94 ± 57.17	0.631
Sagittal R-T angle	57.01 ± 67.00	61.06 ± 74.15	0.758
P wave axis	44.5 ± 25.36	40.35 ± 29.45	0.315
QRS axis	75.78 ± 30.26	69.53 ± 26.57	0.180
T wave axis	34.61 ± 19.69	35.39 ± 17.42	0.797
SV1 (mV)	0.81 ± 0.55	0.76 ± 0.46	0.601
SV5 (mV)	0.47 ± 0.30	0.70 ± 0.50	0.003
RV1 (mV)	0.64 ± 0.33	0.62 ± 0.31	0.646
RV5 (mV)	1.51 ± 0.60	1.46 ± 0.56	0.535

Abbreviations for variables: BMI, body mass index; WBC, white blood cell; Neu%, neutrophil percentage; Lym%, lymphocyte percentage; Mono%, monocyte percentage; Lym, absolute lymphocyte count; Neu, absolute neutrophil count; Mono, absolute monocyte count; RBC, red blood cell; HGB, hemoglobin; PLT, platelet; HCT, hematocrit; RDWC, red cell distribution width; RDWSD, red cell distribution width standard deviation; PDW, platelet distribution width; MPV, mean platelet volume; PLCR, platelet–large contrast ratio; PCT%, procalcitonin percentage; CRP, C-reactive protein; ESR, erythrocyte sedimentation rate; ALT, alanine aminotransferase; AST, aspartate aminotransferase; TB, total bilirubin; DBIL, direct bilirubin; IDIL, indirect bilirubin; ALB, albumin; GLB, globulin; γ-GT, glutamyl transpeptidase; LDH, lactate dehydrogenase; PA, prealbumin; ALP, alkaline phosphatase; UN, urea nitrogen; Crea, creatinine; UA, uric acid; Serum K, serum potassium; Serum Na, serum sodium; Serum Cl, serum chloride; Serum Ca, serum calcium; Serum Mg, serum magnesium; TC, total cholesterol; TG, triacylglycerol; HDLC, high-density lipoprotein cholesterol; LDLC, low-density lipoprotein cholesterol; Apoa, apolipoprotein A; Apob, apolipoprotein B; P wave peak, maximum voltage of P wave among the leads; QRS peak, maximum voltage of P wave among the leads; Frontal I area, area of the I quadrant of the frontal plane VCG; Frontal II area, area of the II quadrant of the frontal plane VCG; Frontal III area, area of the III quadrant of the frontal plane VCG; Frontal IV area, area of the IV quadrant of the frontal plane VCG; Transverse I area, area of the I quadrant of the transverse plane VCG; Transverse II area, area of the II quadrant of the transverse plane VCG; Transverse III area, area of the III quadrant of the transverse plane VCG; Transverse IV area, area of the IV quadrant of the transverse plane VCG; Frontal R-T angle, R-T angle of the frontal plane VCG; Transverse R-T angle, R-T angle of the transverse plane VCG; Sagittal R-T angle, R-T angle of the sagittal plane VCG; RV1, R-wave amplitude in V1; RV5, R-wave amplitude in V5; SV1, S-wave amplitude in V1; and SV5, S-wave amplitude in V5.

**Table 3:** Univariate analysis of risk factors for IVIGR.

Factors	IVIGS (*n* = 187)	IVIGR (*n* = 68)	*p*-Value
Age	3.09 ± 1.99	3.43 ± 2.27	0.247
Male	108	34	0.319
Female	79	34	
BMI (kg/m^2^)	15.99 ± 1.98	15.57 ± 1.77	0.124
***Blood tests***			
WBC (10^9^/L)	13.18 ± 4.15	14.15 ± 4.80	0.115
Neu (10^9^/L)	8.80 ± 3.84	10.72 ± 4.56	0.001
Neu%	66.09 ± 14.91	73.21 ± 16.71	0.001
Lym (10^9^/L)	3.25 ± 2.76	2.27 ± 1.39	0.006
Lym%	24.48 ± 12.28	17.06 ± 11.24	0.001
Mono (10^9^/L)	1.00 ± 1.15	0.78 ± 0.50	0.134
Mono%	6.76 ± 5.04	5.58 ± 2.94	0.071
HGB (g/L)	111.10 ± 10.51	112.05 ± 11.62	0.537
RBC (10^12^/L)	4.23 ± 0.45	4.19 ± 0.44	0.553
RDWC (%)	13.10 ± 1.05	13.00 ± 0.91	0.485
RDWSD (fl)	38.25 ± 2.67	37.90 ± 2.14	0.341
HCT (%)	33.55 ± 2.93	33.47 ± 2.96	0.851
PLT (10^9^/L)	344.03 ± 106.16	331.36 ± 123.23	0.423
PLCR (%)	22.17 ± 7.49	24.07 ± 8.09	0.083
PCT%	0.33 ± 0.09	0.33 ± 0.09	0.569
PDW (fL)	10.51 ± 2.06	10.99 ± 2.21	0.109
MPV (fL)	9.73 ± 0.94	9.97 ± 0.98	0.075
CRP (mg/L)	69.04 ± 42.57	81.75 ± 49.85	0.066
ESR (mm/h)	59.3 ± 27.16	59.72 ± 27.16	0.915
***Biochemistry and lipids***			
ALT (U/L)	58.17 ± 76.81	97.36 ± 172.68	0.077
AST (U/L)	55.61 ± 100.19	77.67 ± 107.32	0.13
GLB (g/L)	25.17 ± 16.32	25.74 ± 8.64	0.786
ALB (g/L)	40.22 ± 4.02	37.55 ± 4.89	0.001
TB (μmol/L)	8.86 ± 9.8	16.28 ± 20.09	0.005
DBIL (μmol/L)	4.00 ± 6.41	10.53 ± 17.48	0.004
IDIL (μmol/L)	4.62 ± 3.03	5.71 ± 3.42	0.017
ALP (U/L)	192.56 ± 62.84	206.21 ± 93.18	0.185
LDH (U/L)	306.22 ± 105.63	309.91 ± 137.98	0.822
Crea (μmol/L)	26.45 ± 6.41	27.89 ± 7.43	0.13
Cystatin C (mg/L)	1.48 ± 7.89	0.82 ± 0.16	0.493
γ-GT (U/L)	50.61 ± 62.39	68.31 ± 82.62	0.07
UN (mmol/L)	3.10 ± 1.14	3.74 ± 1.17	0.001
UA (μmol/L)	215.38 ± 66.75	248.36 ± 78.98	0.001
PA (μmol/L)	58.10 ± 29.11	54.33 ± 22.22	0.122
TC (mmol/L)	3.25 ± 0.51	3.22 ± 0.53	0.757
TG (mmol/L)	1.30 ± 0.38	1.42 ± 0.41	0.039
HDL-C (mmol/L)	0.75 ± 0.23	0.68 ± 0.26	0.033
LDL-C (mmol/L)	2.56 ± 1.47	2.24 ± 0.56	0.087
ApoA (g/L)	0.82 ± 0.20	0.75 ± 0.21	0.018
ApoB (g/L)	0.76 ± 0.14	0.74 ± 0.12	0.323
Serum Na (mmol/L)	135.78 ± 3.04	135 ± 2.82	0.066
Serum Ca (mmol/L)	2.25 ± 0.16	2.21 ± 0.13	0.086
Serum Mg (mmol/L)	0.85 ± 0.08	0.82 ± 0.06	0.035
Serum K (mmol/L)	4.11 ± 0.51	4.11 ± 0.49	0.968
Serum Cl (mmol/L)	101.46 ± 7.95	102.18 ± 3.09	0.470
Serum phosphate (mmol/L)	1.30 ± 0.25	1.27 ± 0.27	0.439
***ECG analysis***			
PR interval (ms)	123.65 ± 15.95	124.03 ± 17.61	0.870
P wave duration (ms)	81.74 ± 11.24	80.93 ± 10.44	0.603
P wave peak (mV)	0.10 ± 0.07	0.09 ± 0.03	0.200
QT interval (ms)	288.68 ± 41.05	280.12 ± 37.23	0.134
Corrected QT interval (ms)	401.01 ± 25.94	400.76 ± 36.27	0.953
QRS duration (ms)	74.22 ± 9.70	77.09 ± 7.83	0.018
QRS peak (mV)	1.67 ± 2.90	1.43 ± 0.53	0.576
Transverse I area	39.47 ± 21.08	40.78 ± 23.93	0.727
Transverse II area	4.20 ± 6.10	2.19 ± 2.91	0.033
Transverse III area	10.12 ± 10.11	9.21 ± 7.62	0.574
Transverse IV area	46.43 ± 24.47	48.21 ± 22.26	0.664
Frontal I area	73.90 ± 18.66	73.39 ± 19.66	0.876
Frontal II area	11.88 ± 13.15	11.48 ± 11.41	0.855
Frontal III area	9.34 ± 10.33	12.10 ± 16.00	0.279
Frontal IV area	5.57 ± 9.72	1.90 ± 2.81	0.012
Frontal R-T angle	22.64 ± 34.58	18.00 ± 39.11	0.451
Transverse R-T angle	43.71 ± 46.83	46.87 ± 41.32	0.685
Sagittal R-T angle	56.29 ± 67.71	61.89 ± 70.32	0.634
P wave axis	43.19 ± 25.59	44.99 ± 28.04	0.632
QRS axis	71.89 ± 29.27	81.79 ± 29.55	0.018
T wave axis	34.36 ± 19.1	35.88 ± 19.63	0.580
SV1 (mV)	0.81 ± 0.57	0.77 ± 0.42	0.598
SV5 (mV)	0.46 ± 0.32	0.62 ± 0.47	0.012
RV1 (mV)	0.66 ± 0.33	0.60 ± 0.33	0.165
RV5 (mV)	1.50 ± 0.62	1.50 ± 0.49	0.977

Abbreviations for variables: BMI, body mass index; WBC, white blood cell; Neu%, neutrophil percentage; Lym%, lymphocyte percentage; Mono%, monocyte percentage; Lym, absolute lymphocyte count; Neu, absolute neutrophil count; Mono, absolute monocyte count; RBC, red blood cell; HGB, hemoglobin; PLT, platelet; HCT, hematocrit; RDWC, red cell distribution width; RDWSD, red cell distribution width standard deviation; PDW, platelet distribution width; MPV, mean platelet volume; PLCR, platelet–large contrast ratio; PCT%, procalcitonin percentage; CRP, C-reactive protein; ESR, erythrocyte sedimentation rate; ALT, alanine aminotransferase; AST, aspartate aminotransferase; TB, total bilirubin; DBIL, direct bilirubin; IDIL, indirect bilirubin; ALB, albumin; GLB, globulin; γ-GT, glutamyl transpeptidase; LDH, lactate dehydrogenase; PA, prealbumin; ALP, alkaline phosphatase; UN, urea nitrogen; Crea, creatinine; UA, uric acid; Serum K, serum potassium; Serum Na, serum sodium; Serum Cl, serum chloride; Serum Ca, serum calcium; Serum Mg, serum magnesium; TC, total cholesterol; TG, triacylglycerol; HDLC, high-density lipoprotein cholesterol; LDLC, low-density lipoprotein cholesterol; Apoa, apolipoprotein A; Apob, apolipoprotein B; P wave peak, maximum voltage of P wave among the leads; QRS peak, maximum voltage of P wave among the leads; Frontal I area, area of the I quadrant of the frontal plane VCG; Frontal II area, area of the II quadrant of the frontal plane VCG; Frontal III area, area of the III quadrant of the frontal plane VCG; Frontal IV area, area of the IV quadrant of the frontal plane VCG; Transverse I area, area of the I quadrant of the transverse plane VCG; Transverse II area, area of the II quadrant of the transverse plane VCG; Transverse III area, area of the III quadrant of the transverse plane VCG; Transverse IV area, area of the IV quadrant of the transverse plane VCG; Frontal R-T angle, R-T angle of the frontal plane VCG; Transverse R-T angle, R-T angle of the transverse plane VCG; Sagittal R-T angle, R-T angle of the sagittal plane VCG; RV1, R-wave amplitude in V1; RV5, R-wave amplitude in V5; SV1, S-wave amplitude in V1; SV5, and S-wave amplitude in V5.

**Table 4:** Incremental predictive value of ECG parameters assessed using LASSO-penalized logistic regression with 10-fold cross-validation.

Outcome/Metric	Laboratory Parameters	Laboratory + ECG Parameters	Δ	*p*-Value
***CAL data***				
AUC	0.744	0.747	0.004	0.897 (DeLong)
NRI (continuous)	—	—	0.098	0.528
NRI for events	—	—	−0.098	0.482
NRI for non-events	—	—	0.196	**0.004**
IDI	—	—	−0.008	0.709
Retained ECG variables	—	Heart rate	0.0004	—
	—	SV5	0.5632	—
***IVIGR data***				
AUC	0.718	0.702	−0.016	0.482 (DeLong)
NRI (continuous)	—	—	0.281	**0.044**
NRI for events	—	—	0.075	0.303
NRI for non-events	—	—	0.206	0.083
IDI	—	—	0.047	**0.002**
Retained ECG variables	—	SV5	−0.049	—

AUC, area under the receiver operating characteristic curve; NRI, net reclassification improvement; IDI, integrated discrimination improvement; CALs, coronary artery lesions; IVIGR, intravenous immunoglobulin resistance. Δ denotes the change (Laboratory + ECG minus Laboratory-only). *p*-values in bold indicate statistical significance (*p* < 0.05).

## Data Availability

All the data are presented in the manuscript. Other datasets used in this study are available from the corresponding author upon reasonable request.
